# GBDR: a Bayesian model for precise prediction of pathogenic microorganisms using 16S rRNA gene sequences

**DOI:** 10.1186/s12864-022-08423-w

**Published:** 2022-03-16

**Authors:** Yu-An Huang, Zhi-An Huang, Jian-Qiang Li, Zhu-Hong You, Lei Wang, Hai-Cheng Yi, Chang-Qing Yu

**Affiliations:** 1 Department of Information Engineering, Xijing University, Xi’an, 710123 China; 2 Center for Computer Science and Information Technology, City University of Hong Kong Dongguan Research Institute, Dongguan, China; 3 College of Computer Science and Software Engineering, Shenzhen University, Shenzhen, 518000 China; 4 Guangxi Academy of Science, Nanning, 530000 China; 5 Xinjiang Technical Institute of Physics and Chemistry, Chinese Academy of Sciences, Ürümqi, 830000 China

**Keywords:** Pathogenic microorganisms, Computational prediction model, 16S rRNA sequence analysis, Microbe-disease association network, Bayesian ranking

## Abstract

**Background:**

Recent evidences have suggested that human microorganisms participate in important biological activities in the human body. The dysfunction of host-microbiota interactions could lead to complex human disorders. The knowledge on host-microbiota interactions can provide valuable insights into understanding the pathological mechanism of diseases. However, it is time-consuming and costly to identify the disorder-specific microbes from the biological “haystack” merely by routine wet-lab experiments. With the developments in next-generation sequencing and omics-based trials, it is imperative to develop computational prediction models for predicting microbe-disease associations on a large scale.

**Results:**

Based on the known microbe-disease associations derived from the Human Microbe-Disease Association Database (HMDAD), the proposed model shows reliable performance with high values of the area under ROC curve (AUC) of 0.9456 and 0.8866 in leave-one-out cross validations and five-fold cross validations, respectively. In case studies of colorectal carcinoma, 80% out of the top-20 predicted microbes have been experimentally confirmed via published literatures.

**Conclusion:**

Based on the assumption that functionally similar microbes tend to share the similar interaction patterns with human diseases, we here propose a group based computational model of Bayesian disease-oriented ranking to prioritize the most potential microbes associating with various human diseases. Based on the sequence information of genes, two computational approaches (BLAST+ and MEGA 7) are leveraged to measure the microbe-microbe similarity from different perspectives. The disease-disease similarity is calculated by capturing the hierarchy information from the Medical Subject Headings (MeSH) data. The experimental results illustrate the accuracy and effectiveness of the proposed model. This work is expected to facilitate the characterization and identification of promising microbial biomarkers.

**Supplementary Information:**

The online version contains supplementary material available at 10.1186/s12864-022-08423-w.

## Background

Researchers are increasingly aware of the critical effects of the human microorganisms on our physical condition. Microorganisms, a.k.a. microbes, are referred to viruses, bacteria, archaea, and eukaryotes (protozoa and fungi) [1]. They can inhabit and thrive in almost each kind of natural environments, of course including the human body. Human microbial communities locate in different parts of the human body, including the external (e.g. skin) and the internal (e.g. the mucosal epithelia of vagina and intestine). In the adult gut, the large majority of intestinal microbes (10^13^–10^14^) inhabiting the gastrointestinal tract can approach the population quantity of human cells [2]. Recently, accumulating evidences [3–5] show that the onset of human disorders could be attributed to the dysfunction of human microbiota.

The symbiotic relationship between the human microbiota and its host has been demonstrated to get involved in multiple important biological activities. The human microbiota can be influenced by multiple factors of its host such as the genetics, lifestyle, body site, age, health status and others (e.g. antibiotics and smoking) [6–9]. The resident microbial flora can also affect human physical conditions via multiple microbial genome-encoded metabolic functions. Such metabolic functions can strengthen the metabolic capacity of its host. Therefore, human microbes play a key role in many important biological processes, e.g., by defending against pathogens, enhancing the immune system, getting access to nutrients, as well as degrading toxic compounds [6]. Identifying the latent relationships between microbes and human diseases can provide valuable insights into understanding the pathology of human diseases. For example, butyrate as the primary energy source of intestinal epithelial cells can also function as a key component to suppress the signal transduction pathways of expressing proinflammatory cytokines. Individuals with inflammatory bowel disease (IBD) have been found to have population declines of butyrate-producing microbes, such as *Clostridium leptum* and *Clostridium coccoides* groups [10]. This phenomena could also lead to decreasing butyrate utilization [11], which implies the fact that the restoration of host-microbe equilibrium could cure or prevent human complex diseases.

Thanks to the high volume of genomic data by high-throughput techniques, increasing bioinformatics tools and databases have been proposed for downstream analysis and data management [12–14]. For examples, a 16S rDNA analysis toolkit named W.A.T.E.R.S [15]. can be used for sequence alignment, operational taxonomic units (OTUs) determination, phylogenetic tree construction, and etc. Moreover, hundreds of microbe-disease associations are publicly available in Human Microbe-Disease Association Database (HMDAD) [16]. However, the known microbe-disease associations are just the tip of the iceberg and far from enough towards a complete picture for clinical medicine. The routine wet-lab experiments for biomarker discovery are easy to fail in clinical trials after considerable effort, money, and time have been already invested. In recent years, computational models have been proposed to prioritize seminal biomarker candidates using heterogeneous biological information in several fields including risk gene-disease association prediction [17–19], protein-protein interaction (PPI) prediction [20, 21], drug-target interaction prediction [22], and etc. [14]. The successful applications of these studies motivate us to devise an effective computational model for prioritizing potential pathogenic microbes.

16S ribosomal RNA (rRNA) gene encodes the 30S small subunit of the ribosomal RNA molecules of ribosomes. The low resolution of 16S rRNA gene enables the rapid and accurate identification to establish taxonomic relationships between microbes [23]. The major difference between 16S rRNA gene sequences (~ 1500 bp) tends to fall in the nine hypervariable regions (V1-V9), representing dramatic variations for alignments. Therefore, 16S rRNA sequencing analysis is widely used to capture natural species-specific “fingerprints” for phylogenetic comparisons.

Recently, increasing effective computational prediction models like KATZHMDA [24] and PBHMDA [25] were developed to explore the potential microbe-disease associations using the known microbe-disease association network as well as the calculated homologous similarity matrices. In light of a social network prediction algorithm called KATZ [26], KATZHMDA proposes a new proxy measure index to calculate the probabilities of unknown microbe-disease associations by considering the number of walks within the network and their own lengths. Moreover, PBHMDA is developed as a path-based prediction model to perform a restricted depth-first search by traversing all possible paths between microbes and diseases. However, some limitations could limit their usage and effectiveness, for example, by introducing the systematic bias of the predicted similarity matrices, merely focusing on the known diseases and microbe, and applying the global scoring schemes. Under the hypothesis that the functionally similar microbes tend to share the similar interaction patterns with pathologically similar human diseases, the ultimate goal of this work is to facilitate the discovery of validated biomarkers for helping the early diagnosis, risk assessment, tracking progression, and drug development. Here, we present a Group based computational model of Bayesian Disease-oriented Ranking (GBDR) for identification of potential microbe-disease associations based on the HMDAD database. Heterogeneous biological information is leveraged to compute similarity matrices, including disease semantic similarity, microbe similarity based on BLAST+ scores and microbe similarity based on MEGA7 evolutionary distance scores. The proposed model obtains the supreme prediction accuracy via leave-one-out cross validation (LOOCV) and *k*-fold cross validation (*k*-fold CV) in comparison with other state-of-the-art methods. Experiment results demonstrate that the group-based collaboration filtering and inferred similarity matrices can contribute to the improvement of prediction performance. We conduct a case study for an important disease to manually validate those predicted pathogenic microbes ranked in the top-20 list via published literatures. As a result, the reliable performance of the proposed model is fully demonstrated. It is anticipated that GBDR could be an effective computational tool to accelerate the identification of pathogenic microorganisms.

## Results

### Cross validation and case study

Under the frameworks of LOOCV and k-fold CV, the performance of GBDR is thoroughly evaluated. Since GBDR is devised as a disease-oriented ranking computational model, it aims to prioritize the most potential microbes for each disease. As such, we adopt a local scoring scheme for performance evaluation. As for LOOCV, each known microbe-disease association is used to test the model in turns while the rest are used for training until all counterparts are selected. Similarly, in the simulations of *k*-fold CV, the whole set of known microbe-disease associations are randomly split into *k* groups, where (k-1) groups form a training set while the remainder is a testing set until each group is tested in truns. To reduce the bias of random divisions, 50 times *k*-fold CVs are conducted to then achieve the average results.

The microbe-disease association prediction is actually a binary classification problem. The receiver operating characteristic (ROC) curve is extensively used to evaluate the performance of binary classification models. It is plotted by the true positive rate (sensitivity) versus false positive rate (1-specificity). In this work, sensitivity/specificity represents what a high probability of a predicted result can be told to make a positive/negative prediction correctly. The area under ROC curve (AUC) is a numerical evaluation coefficient between 0 and 1. For a disease *d*, the AUC value can be defined accordingly as:

1 $${\mathrm{AUC}}_d=\frac{1}{\left|{\mathrm{\mathcal{R}}}^{te}(d)\right|}{\sum}_{\left(i,j\right)\in {\mathrm{\mathcal{R}}}^{te}(d)}\delta \left({\hat{r}}_{di}>{\hat{r}}_{dj}\right)$$

where ${\mathcal{R}}^{te}(d)=\left\{\left(i,j\right)|\left(d,i\right)\in {\mathcal{R}}^{te},\left(d,j\right)\notin \mathcal{R}\cup {\mathcal{R}}^{te}\right\}$. ${\mathcal{R}}^{te}(d)$ is a test dataset of *d*, ${\hat{r}}_{di}$ and ${\hat{r}}_{dj}$ are predicted values, and *δ*() is a binary indicator. If the equation within the brackets is true, *δ*() =1, otherwise 0. The final AUC value can be averaged as follows:

2 $$\mathrm{AUC}=\frac{\sum_{u\in {D}^{te}}{AUC}_d}{\left|{D}^{te}\right|}$$

Here, *D*^*te*^ is a disease set on testing sets. Normally, AUC = 1 represents a perfect prediction and AUC = 0.5 represents a completely random one.

**Fig. 1:**
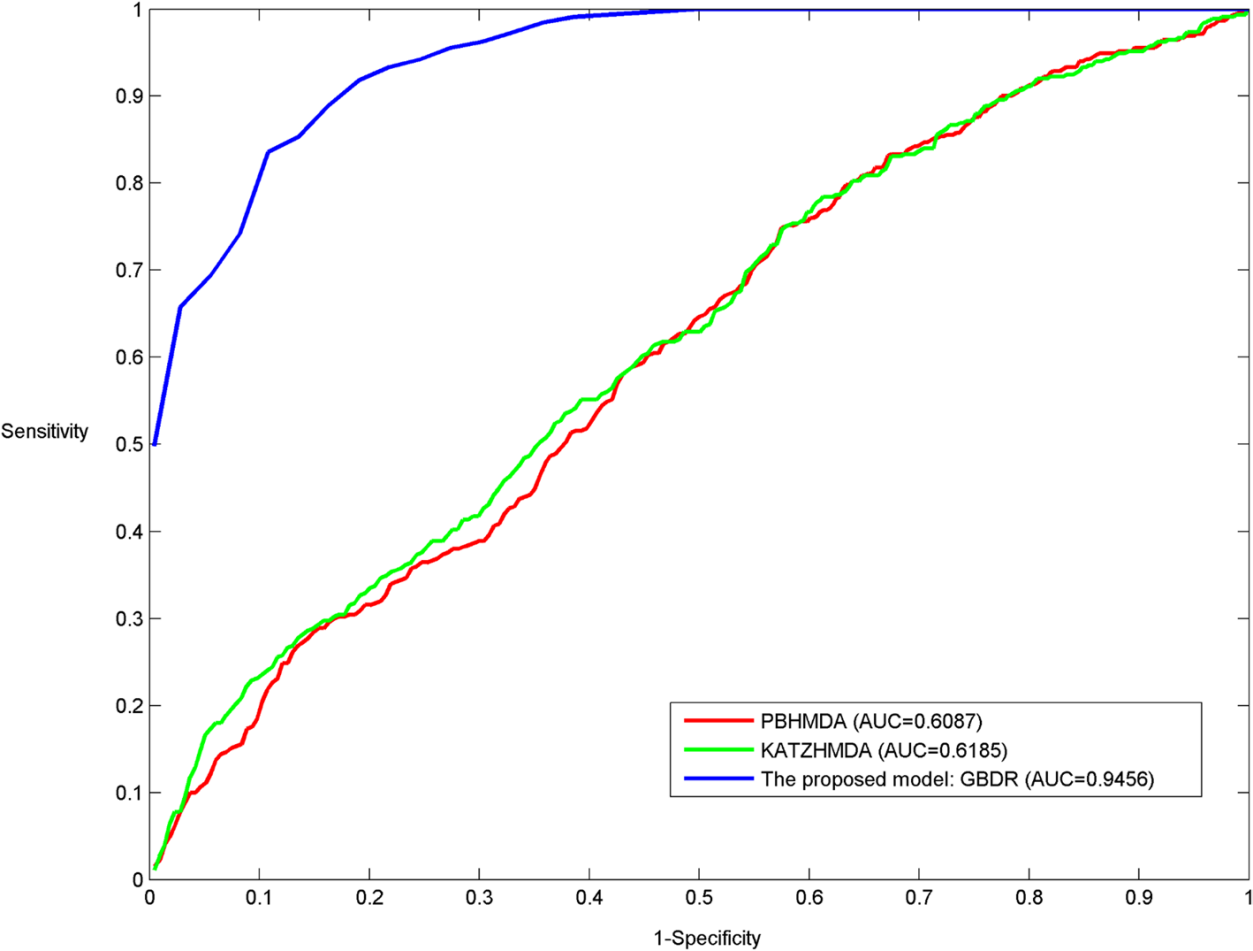
The proposed model is compared with PBHMDA and KATZHMDA based on HMDAD database via LOOCV

First of all, GBDR is compared with PBHMDA and KATZHMDA based on the known microbe-disease associations from HMDAD database via LOOCV (see Fig. 1). For a fair comparison, all compared models employ the same data resources, i.e. disease semantic similarity, microbe similarity based on BLAST+ scores and microbe similarity based on MEGA7 evolutionary distance scores. GBDR, PBHMDA and KATZHMDA achieve AUC values of 0.9456, 0.6087 and 0.6185, respectively. The proposed model performs better than the other two state-of-the-art models. PBHMDA and KATZHMDA have similar prediction performance in terms of local LOOCV. Since both of them are proposed to globally predict the most potential microbe-disease associations using the global scoring schemes, the class imbalance could lead to degrade their prediction performance to some extent.

**Fig. 2:**
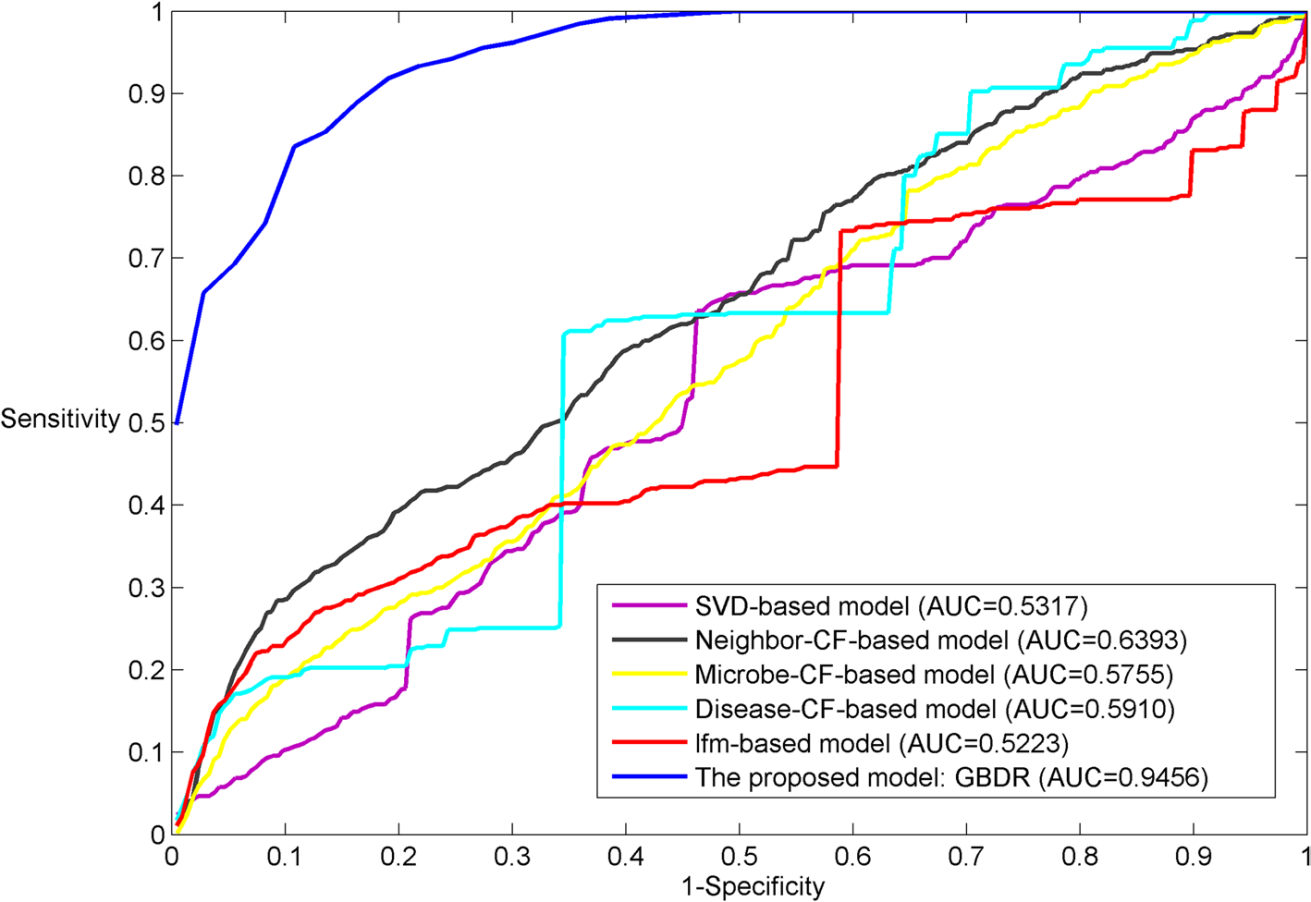
The comparison result between GBDR and other recommendation algorithms via LOOCV

Second, the proposed model is also compared with the other representative algorithms of recommender system via LOOCV (see Fig. 2), including singular value decomposition (SVD) based model, latent factor model (LFM), microbe-based collaborative filtering (CF), disease-based CF and neighbor-based CF models. Since GBDR is originally proposed as a recommendation algorithm, the fundamental assumption of pairwise association is adopted to resolve the limitations of the pointwise association assumption where the unknown (unlabeled) microbe-disease association are irrelevant. It is interesting to know the performance difference between the GBDR and other representative recommendation algorithms. Since the purpose of this work is to “recommend” the most possible microbes to a certain disease, it is intuitive and meaningful to use these recommendation algorithms for the performance comparison. As we can see in Fig. 2, GBDR also achieves the highest AUC of 0.9456. These representative recommendation algorithms tend to show a moderate predictive power in this case. Among these classical recommendation algorithms, the neighbor-based CF model obtains the best performance achieving the AUC of 0.6393. The result suggests that the disease-oriented ranking model with Bayesian filtering is capable of capturing latent relationships between microbes and diseases. The new and improved assumption in Bayesian disease-oriented ranking is more effective for prediction by introducing richer interactions among microbes. Particularly, the unified effect of group preference and individual preference is linearly combined to naturally maximize the overall likelihood.

Finally, we also implement *k*-fold CV for further evaluation (see Table 1). As a result, the proposed model yields average AUCs of 0.8266 ± 0.0805, 0.8866 ± 0.0270 and 0.8926 ± 0.0167 in 2-fold CV, 5-fold CV and 10-fold CV, respectively. Both LOOCV and *k*-fold CV can demonstrate the effectiveness of GBDR. Furthermore, colorectal carcinoma (CRC) is selected as an important human disease for a case study. As a result, 9 out of top-10 and 16 out of top-20 predicted microbes have been experimentally confirmed to have associations with the development of CRC. Detailed information is provided in Additional file 1.

**Table 1:** The proposed model is evaluated in 2-fold, 5-fold and 10-fold CV, respectively

	2-fold CV	5-fold CV	10-fold CV
AUC	0.8266+/− 0.0805	0.8866+/− 0.0270	0.8926+/− 0.0167

### Effectiveness evaluation of group-based collaboration filtering

**Fig. 3:**
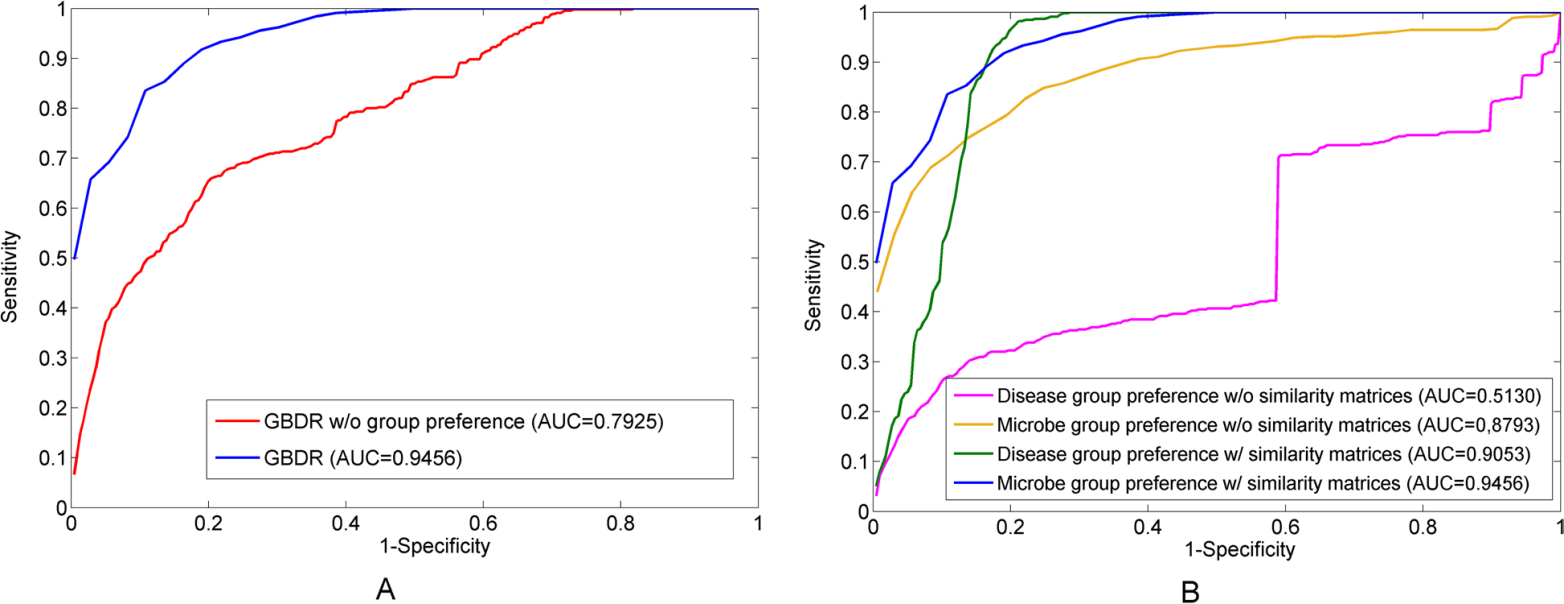
The prediction performance with and without the group preference is evaluated by LOOCV in part **A**. And the proposed model based on disease group preference or microbe group preference is evaluated by LOOCV respectively in part **B**

In this section, we conduct LOOCV to evaluate the prediction performance with or without the group-based preference strategy (as shown in Fig. 3A). Without the group preference, the proposed model suffers a nearly 16.2% decline in prediction accuracy with an AUC value of 0.7925. It shows that the group preference strategy is efficient to aggregate the group preference for the disease-oriented ranking through injecting richer interactions among microbes. The linear combination of pairwise preference and group preference is more effective than the simple pairwise preference.

Moreover, we further evaluate the prediction performance of the proposed model with the disease-based or microbe-based group preference respectively via LOOCV (see Fig. 3B). Firstly, without integrated similarity of microbe or disease used in Eq. (18), the proposed model only leverages the known microbe-disease associations for prediction. In this scenarios, combined with microbe-based group preference, the proposed model obtains an improved performance with AUC of 0.8793. And the counterpart with disease-based group preference yields an AUC value of 0.5130. This result supports our assumption that the coordinated functions of microbial groups may pathologically influence the susceptibility to human diseases whereas the human diseases fail to have a significant group trend to affect microbial communities. Secondly, based on the known microbe-disease associations, the proposed model is carried out with both microbe and disease similarity matrices using the microbe-based group preference. In this way, the proposed model achieves the best prediction performance with AUC of 0.9456. On the other hands, the proposed model with the disease-based group preference shows a significant increase (39.23%) in the prediction accuracy achieving the AUC of 0.9053. This result suggests that the inferred similarity matrices provide useful heterogeneous information to effectively discriminate seminal biomarker candidates.

### Effectiveness evaluation of combining different types of similarities

As mentioned at the end of the above section, the inferred similarity matrices can effectively improve the prediction performance of our model based on the disease-based group preference. It motivates us to conduct the performance effect analysis of different types of similarities proposed in our model via 5-fold CV. The results are shown in Table 2. As a baseline, the GBDR without using any similarity matrices shows the average AUC of 0.6189 with standard deviation of 0.0561. Using the disease semantic similarity, the prediction accuracy achieves average AUC value of 0.6796+/− 0.0468 with 6.27% improvement. Moreover, when the GBDR is integrated with both microbe similarity based on BLAST+ scores and microbe similarity based on MEGA7 evolutionary distance scores, the prediction accuracy is improved by 10.02% achieving the average AUC value of 0.7171+/− 0.0427. Finally, the GBDR obtains the best average AUC value of 0.8081+/− 0.0284 using the disease semantic similarity and the integrated microbe similarity. The result demonstrates that the sequence information of gene exploited by computational approaches (BLAST+ and MEGA 7) enables the precise measure of the biological homology between microbes. Furthermore, the disease semantic similarity based on the Medical Subject Headings (MeSH) descriptors can reflects the molecular relatedness between hereditary diseases. Although the known microbe-disease association network is sparse as its links are limited in number, applying diverse similarities to the proposed model is useful to provide discriminative biological information, and therefore enabling the precise prediction of pathogenic microbes.

**Table 2:** When combined with different similarity matrices, the proposed model is evaluated via 5-fold CV based on the disease-based group preference

Combined similarity matrices	5-fold CV
No integrated similarity of microbe or disease via Eq. (18)	0.6169+/− 0.0561
Disease semantic similarity	0.6796+/−0.0468
Microbe sequence similarity and microbe evolutionary distance-based similarity	0.7171+/−0.0427
Disease semantic similarity, microbe sequence similarity and microbe evolutionary distance-based similarity	0.8081+/−0.0284

### Effect analysis of key parameters in GBDR

**Fig. 4:**
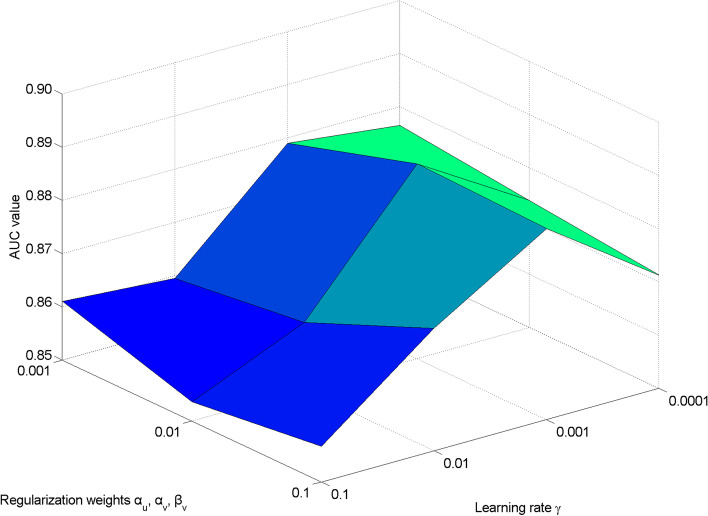
The parameter analysis of regularization weight *α*_*u*_, *α*_*v*_ and *β*_*v*_ versus learning rate γ

**Fig. 5:**
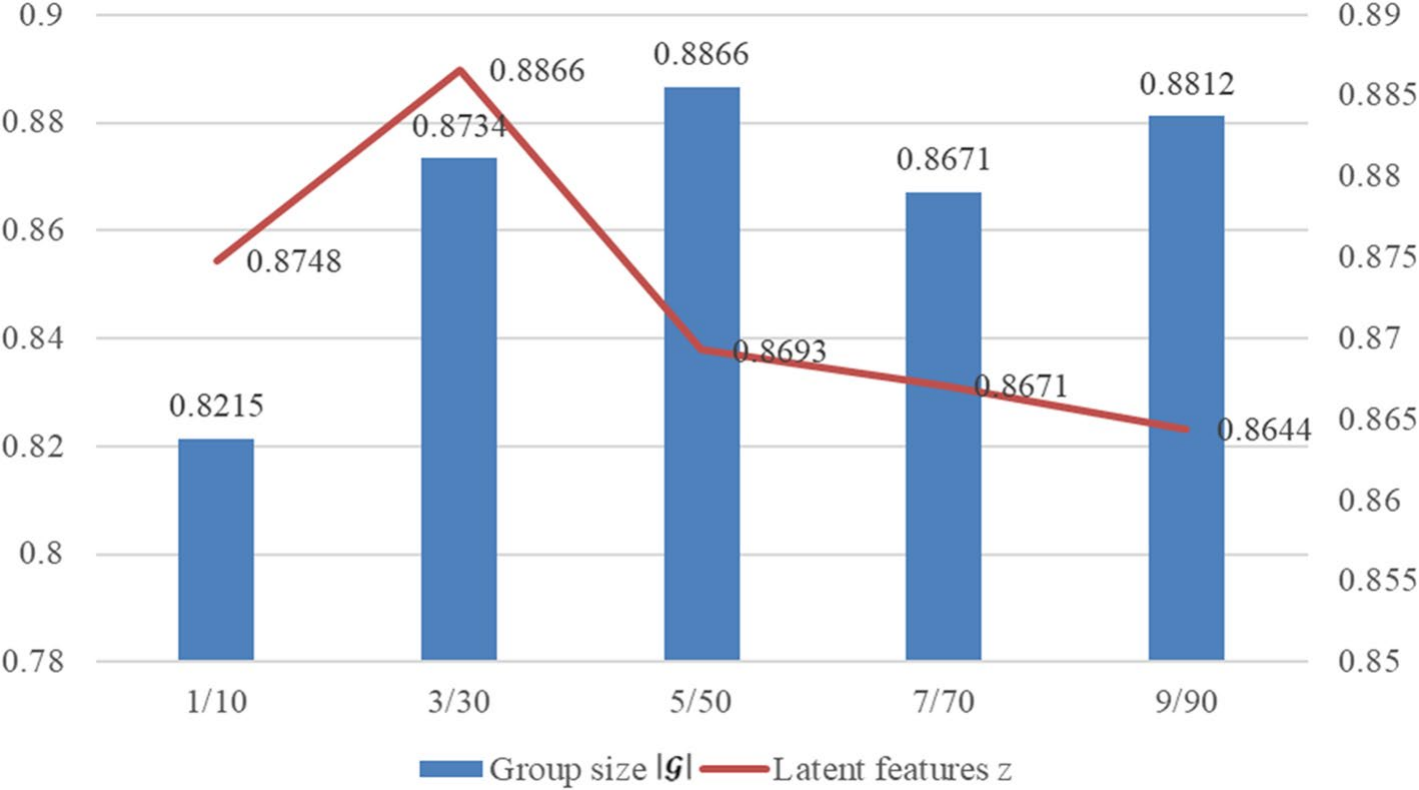
The effect influence of group size $\left|\mathcal{G}\right|$ and number of latent features z

There are several key parameters in GBDR, e.g., regularization weights ***α***_***u***_, ***α***_***v***_ and ***β***_***v***_, learning rate **γ**, number of later features z, and group sizes $\left|\mathbf{\mathcal{G}}\right|$. Based on 5-fold CV, we conduct an effect analysis to explore the potential of parameter tuning. As we can see in Fig. 4, GBDR reaches the peak of AUC values when regularization weights ***α***_***u***_, ***α***_***v***_ and ***β***_***v***_ are to 0.01 and learning rate **γ** is set to 0.001. By evaluating all possible combinations via grid search, the achieved AUC values vary from 0.8536 to 0.8866. For better demonstration, we combine the results of z and $\left|\mathbf{\mathcal{G}}\right|$ as a whole in Fig. 5. Regarding of the different number of latent features z, no significant change is observed in terms of AUC values. Based on the results, we set 30 as the default value of z. Moreover, GBDR achieves the highest AUC value when $\left|\mathbf{\mathcal{G}}\right|$ is set to 5. As a baseline, GBDR without group preference setting (i.e., $\left|\mathbf{\mathcal{G}}\right|$ =1) suffers from degradation by 6.5% as expected. In short, the performance of GBDR is not sensitive to the key parameters tuning.

## Discussion

Accumulating evidence show that different types of microbiota are associated with the mechanism of human diseases, forming a complex causal network. Although there are a number of methods having been proposed for predicting such important associations, 16 s rRNA gene sequences, the information most easily obtained in microbe research, haven’t been utilized for this task. To bridge this gay, we proposed a Bayesian prediction model called GBDR, using various types of information including 16 s rRNA sequences. GBDR is based on the computation of disease/microbe similarity assuming that similarity microbes tend to be involved in similar disease mechanism. The experimental results show that the prediction based on such an assumption is feasible and effective. We anticipate that GBDR can help the researchers find the relevant diseases for a specific type of microbe given its 16 s rRNA sequence.

## Conclusion

The human microbiota has attracted the increasing attention thanks to its key role playing in human biological activities. It has even been deemed as the “forgotten organ” in the human body. Recent researches show the dysfunction of host-microbiota homeostatic balance can result in the onset of various human diseases. The great advance of technology, especially PCR amplification and next-generation sequencing, allows to generate high volumes of sequences, providing a new window for the follow-up downstream analysis. In this work, we leveraged 16S rRNA gene to infer microbe similarity based on BLAST+ scores and microbe similarity based on MEGA7 evolutionary distance scores. The framework of GBDR is proposed to identify the most seminal disease-specific microorganisms on a large scale. Based on the results of simulation experiments, GBDR is demonstrated to achieve higher performance than the two state-of-the-art models and representative recommendation algorithms via LOOCV. Furthermore, reliable predictive capability of GBDR is validated by *k*-fold CV and a case study. GBDR is expected to provide valuable insights into advancing the identification of potential microbes as ideal biomarkers for evaluating and measuring human complex diseases. The prediction list of the most seminal pathogenic microbes is released in Additional file 2 sorted by various specific diseases. Based on the assumption that functionally similar microbes tend to share the similar interaction patterns with human diseases, the main goal of this work is to prioritize the most potential disease-related microbes. That is, the involved microbes could be affected by the involved human diseases and/or the human diseases could be caused by the involved microbes. Identifying the seminal disease-microbe association is the first key step to develop the full potential for further in vitro tests in therapeutics and clinical research.

Several factors can be summarized to improve the effectiveness of GBDR. Firstly, the integrated microbe similarity holds a significant potential to characterize the remarkable feature patterns between microbes. The hierarchical relevance is leveraged to measure disease semantic similarity. Secondly, the group-based pairwise strategy is capable of extracting the fruitful information based on the group preference of microbes associating with a certain disease. Thirdly, the Bayesian approach with disease-oriented ranking is quite suitable for the local prediction of microbe-disease associations. However, GBDR adopts a local scoring scheme for disease-oriented ranking without global normalization process. GBDR is inapplicable to globally predicting the most potential microbe-disease associations like PBHMDA and KATZHMDA.

## Methods

### Materials

In this work, three types of biological information are utilized, i.e. the known microbe-disease associations derived from HMDAD database (http://www.cuilab.cn/hmdad) **[**16**]**, 16S rRNA partial or complete gene sequences downloaded from the Nucleotide Database of the National Centre for Biotechnology Information (NCBI) **[**27**]** and MeSH descriptors provided by the Nation Library of Medicine (NLM) **[**28**]**. It is noted that, Ma et al. **[**16**]** searched articles regarding human microbiome-related research published before July 2014. The HMDAD database provides 450 non-repetitive known microbe-disease associations including 292 microbes and 39 human diseases (see Additional file 3). The numbers of microbes and diseases are denoted as *nm* and *nd*, respectively. We note that all the symbols that used in the Methods section are summarised in the Table 3. All known microbe-disease associations are converted into an adjacency binary matrix as variable $\mathbf{\mathcal{R}}$ of size nm × nd representing their association relationships. Namely, $\mathbf{\mathcal{R}}\left({\boldsymbol{m}}_{\boldsymbol{i}},{\boldsymbol{d}}_{\boldsymbol{j}}\right)=\mathbf{1}$ indicates microbe *mi* is known to be associated with disease *dj*, otherwise $\mathbf{\mathcal{R}}\left({\boldsymbol{m}}_{\boldsymbol{i}},{\boldsymbol{d}}_{\boldsymbol{j}}\right)=\mathbf{0}$.

**Table 3:** Summary of the symbols used

# of microbes: *nm*	# of diseases: *nd*
known microbe-disease associations: $\mathbf{\mathcal{R}}$	# of latent features: *z*
Disease similarity: *S*_*d*_	Microbe similarity: *S*_*m*_
Predicted probability: $\hat{\boldsymbol{r}}$	Bernoulli distribution: **δ** ()
Group preference: $\mathbf{\mathcal{G}}$	Objective function: $\mathbf{\mathcal{F}}$
Regularization weights: ***α***_***u***_, ***α***_***v***_ and ***β***_***v***_	Microbe latent feature vector: ***U***
Disease latent feature vector: V	Bias value: ***b***
Model parameters: **Θ**	Learning rate: **γ**

### Disease semantic similarity

**Fig. 6:**
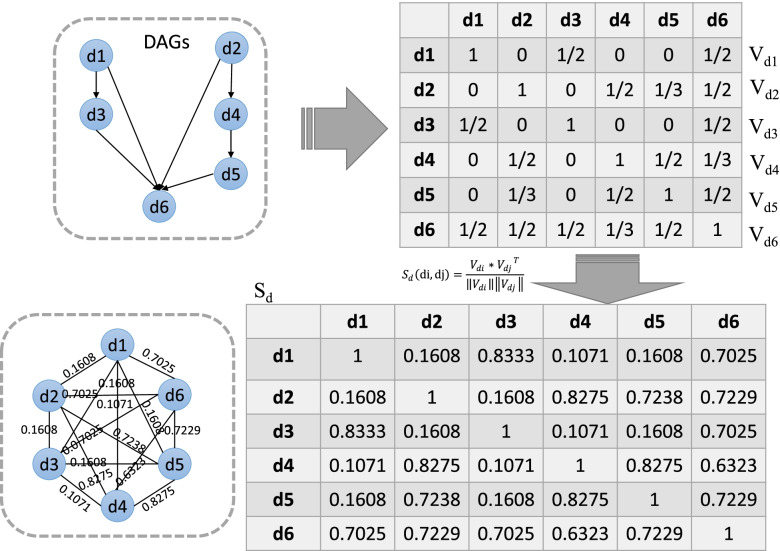
The calculation process of disease semantic similarity *S*_*d*_

The hierarchy system of MeSH descriptors is informative to offer semantic-based taxonomic categorization for various human diseases. For example, the MeSH ID of overnutrition (C18.654.726) shares the same prefix with its subtype obesity’s (C18.654.726.500). Accordingly, the relationships between any disease and others can be established by respective Directed Acyclic Graphs (DAGs) using the hierarchy of MeSH IDs [29]. Each disease has at least one MeSH ID which numerically represents its location in DAGs. Figure 6 illustrates the calculation process of disease semantic similarity. Empirically, the shorter path between the ancestor node *d* and the target node *t*, the higher weight value should be given. It can be formulated as follows:

3 $${V}_d(t)=\left\{\begin{array}{c}1,\kern7.5em if\ t=d\\ {}\frac{1}{len\left(d,t\right)+1},\kern1.25em if\ t\in \left\{ the\ descendant\ node\ of\ d\right\}\\ {}0,\kern7.25em otherwise\end{array}\right.$$

where *len*(*d*, *t*) is the shortest path length between the ancestor node *d* and one of its descendant node *t*. For example in Fig. 6, *d*_*1*_ is the ancestor node of *d*_*3*_ and *d*_*6*_. *V*_*d*1_(*d*_3_) = 1/(*len*(*d*_1_, *d*_3_) + 1) = 1/2 where *len*(*d*_1_, *d*_3_) = 1. Likewise, *V*_*d*1_(*d*_6_) = 1/(*len*(*d*_1_, *d*_6_) + 1) = 1/2 where *len*(*d*_1_, *d*_6_) = 1. In this way, a feature vector for *d*_*1*_ can be computed as *V*_*d*1_ = (1, 0, 1/2, 0, 0, 1/2). Then, we further calculate the semantic similarity of any two diseases *d*_*i*_ and *d*_*j*_ by cosine similarity measure:

4 $${S}_d\left(\mathrm{di},\mathrm{dj}\right)=\frac{V_{di}\ast {V_{dj}}^T}{\left\Vert {V}_{di}\right\Vert \left\Vert {V}_{dj}\right\Vert }$$

where *V*_*di*_ and *V*_*dj*_ are the feature vectors of *d*_*i*_ and *d*_*j*_, respectively.

### Microbe similarity based on BLAST+ scores and MEGA7 evolutionary distance scores

Sequence similarity and evolutionary distance-based similarity are two effective measurements to examine the relatedness among microbes from different perspectives. The former reflects the degree of likeness between any two sequences while the latter refers to the divergence of their common ancestral sequence. Although the calculation of both are based on the same information source, i.e., 16S rRNA gene sequences, they do not have to be similar as a necessary condition. Basic Local Alignment Search Tool (BLAST) is a specific sequence similarity search program (http://www.ncbi.nlm.nih.gov/blast) [30]. We use its variant BLAST+ [31] to compare a targeted 16S rRNA gene sequence of each target microbe against the sequences of other microbes as a nucleotide sequence database in turns. *Identity* is an important glossary of BLAST+ to measure the extent to which two (nucleotide or amino acid) sequences are invariant in an alignment. In this work, *identity* is used for measuring microbe sequence similarity. In this way, we define a matrix as *Iden* of size *nm*×*nm* to store the *identity* values yielded by the alignment. Then the microbe sequence similarity denoted as *MSS* is calculated by normalizing *Iden* matrix as follows:

5 $$\mathrm{MSS}\left(\mathrm{mi},\mathrm{mj}\right)=\frac{Iden\left( mi, mj\right)-\mathit{\operatorname{Min}}.(Iden)}{\mathit{\operatorname{Max}}.(Iden)-\mathit{\operatorname{Min}}.(Iden)}$$

It is noted that, among 292 investigated microbes, five microbe have no available 16S rRNA gene sequences in NCBI (denoted as “unavailable” to “FASTA filename” in Additional file 3). We simply set their sequence similarities as the mean of the rest available.

The calculation of microbe evolutionary distance-based similarity is mainly based on the molecular evolutionary genetics analysis of MEGA 7 [32] (http://www.megasoftware.net/). The evolutionary distance between any two sequences is measured by the number of nucleotide substitutions involved. First, Clustal W is used to perform multiple sequences alignment [33]. To reduce the disturbance caused by the gaps, all sequences are trimmed down to the shortest size by removing terminal redundancy at 5′ and 3′ terminus. Then the option of complete deletion option is set to address the issues of gaps and missing data. We utilized *p*-distance model to measure the evolutionary distances based on substitutions (including transitions and transversions). *p*-distance [34] for nucleotide sequences is written as:

6 $$\hat{p}=\frac{n_d}{n}$$

where *n*_*d*_ refers to the number of different nucleotides between two tested sequences and *n* is the total number of nucleotides examined. The higher value of evolutionary distances denotes the higher evolutionary diversity. The evolutionary distances are subtracted from 1 and the result is denoted by a matrix as *ED* of size *nm*×*nm*. Similarly, the microbe evolutionary distance-based similarity (*MES*) is also normalized as follows:

7 $$\mathrm{MES}\left(\mathrm{mi},\mathrm{mj}\right)=\frac{ED\left( mi, mj\right)-\mathit{\operatorname{Min}}.(ED)}{\mathit{\operatorname{Max}}.(ED)-\mathit{\operatorname{Min}}.(ED)}.$$

For those microbes without available 16S rRNA gene sequences, their evolutionary distance-based similarities are also set to the overall mean level. If the unavailable microbe list increases, performance degeneration is inevitable to happen. We can address this problem based on the know microbe-disease associations by exploiting the implicit information from the topological network structure. According to the previous works [22, 35], Gaussian interaction profile kernel similarity and local similarity-based methods (e.g., the Jaccard index and Salton index) can be applied to calculate the biological function-based similarity of those microbes without available 16 s rRNA gene sequences. Finally, we empirically merge *MSS* and *MES* to represent the final microbe similarity *S*_*m*_:

8 $${S}_m\left({m}_i,{m}_j\right)=\frac{MSS\left({m}_i,{m}_j\right)+ MES\left({m}_i,{m}_j\right)}{2}$$

### Group preference based Bayesian disease-oriented ranking

**Fig. 7:**
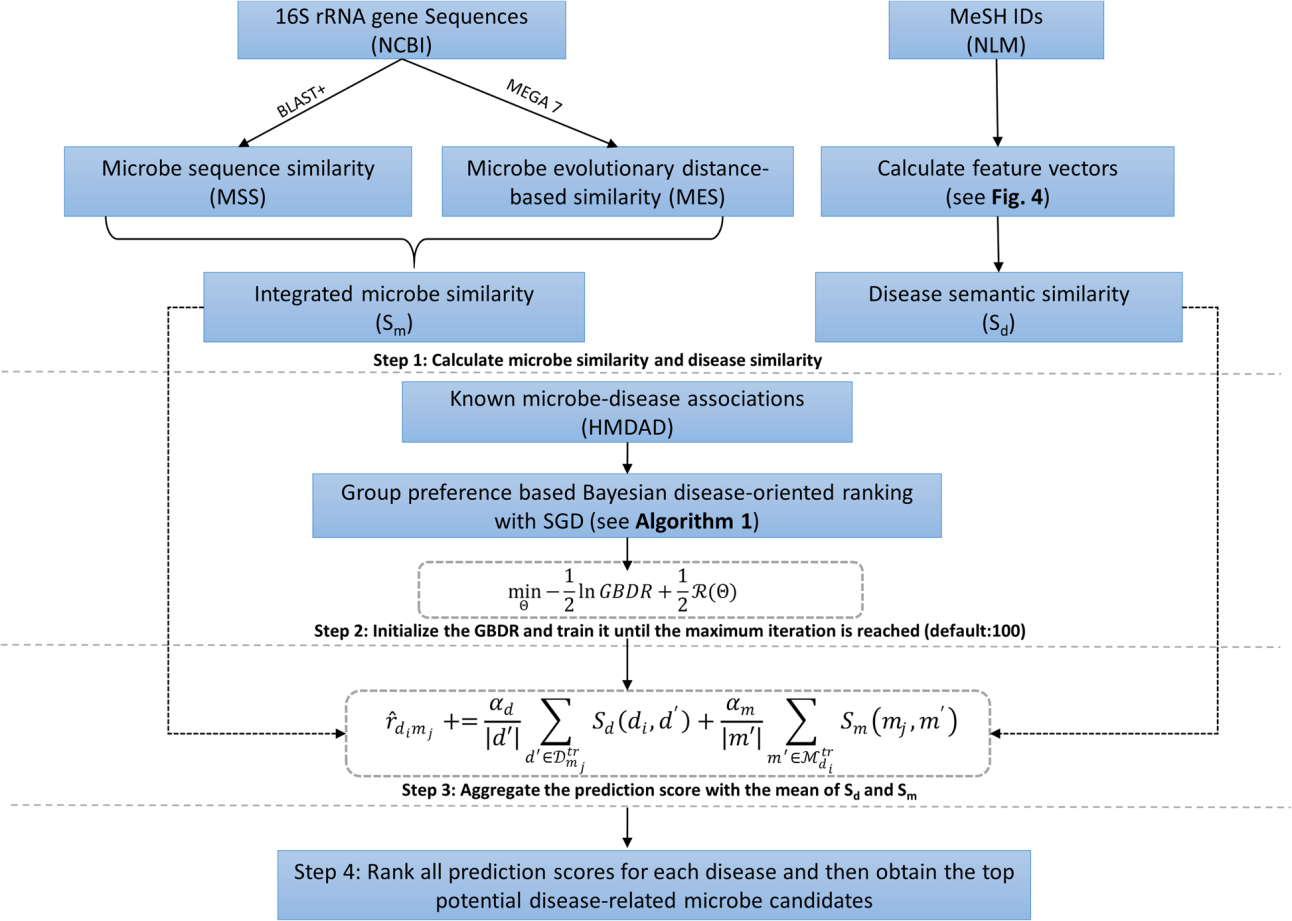
The flowchart of GBDR

Based on the previous work [36, 37] in recommender system, the pointwise association assumption, i.e., considering all known microbe-disease associations as “interactions” and unknown ones as “no interactions”, could mislead the learning process. However, the pairwise association assumption over two microbes could relax the pointwise preference assumption by treating that a disease *d* is more probably related to a microbe *i* than a microbe *j* represented as ${\hat{\boldsymbol{r}}}_{\boldsymbol{di}}>{\hat{\boldsymbol{r}}}_{\boldsymbol{dj}}$ where *i* belongs to the known association with *d* whereas *j* does not. Empirically, this assumption generates better prediction results than the pointwise assumption. Inspired by this idea [38, 39], we present group pairwise preference based Bayesian disease-oriented ranking for prioritizing the most potential pathogenic microbes (see Fig. 7).

Based on the known microbe-disease associations for a typical disease *d*, we first define the overall likelihood of pairwise preferences (LPP) among the whole set of microbe (denoted as $\mathbf{\mathcal{M}}$**)**:

9 $${\displaystyle \begin{array}{c}\mathbf{LPP}\left(\boldsymbol{d}\right)={\prod}_{\boldsymbol{i},\boldsymbol{j}\in \mathbf{\mathcal{M}}}\Pr {\left({\hat{\boldsymbol{r}}}_{\boldsymbol{di}}>{\hat{\boldsymbol{r}}}_{\boldsymbol{dj}}\right)}^{\boldsymbol{\delta} \left(\left(\boldsymbol{d},\boldsymbol{i}\right)\succ \left(\boldsymbol{d},\boldsymbol{j}\right)\right)}\times {\left[1-\Pr \left({\hat{\boldsymbol{r}}}_{\boldsymbol{di}}>{\hat{\boldsymbol{r}}}_{\boldsymbol{dj}}\right)\right]}^{\left[1-\boldsymbol{\delta} \left(\left(\boldsymbol{d},\boldsymbol{i}\right)\succ \left(\boldsymbol{d},\boldsymbol{j}\right)\right)\right]}\\ {}={\prod}_{\left(\mathbf{d},\mathbf{i}\right)\succ \left(\mathbf{d},\mathbf{j}\right)}\Pr \left({\hat{\boldsymbol{r}}}_{\boldsymbol{di}}>{\hat{\boldsymbol{r}}}_{\boldsymbol{dj}}\right)\left[1-\Pr \left({\hat{\boldsymbol{r}}}_{\boldsymbol{di}}>{\hat{\boldsymbol{r}}}_{\boldsymbol{dj}}\right)\right]\end{array}}$$

where (***d***, ***i***) **≻** (***d***, ***j***) means that disease *d* is more potentially associated with microbe *i* than microbe *j*. And **δ**((***d***, ***i***) **≻** (***d***, ***j***)) is Bernoulli distribution over the binary random variable. To better approximate the disease-oriented pairwise preference over two microbes, the Bayesian disease-oriented ranking method is adopted to simplify the term **LPP**(***d***) as follows [38]:

10 $$\mathbf{BDR}\left(\boldsymbol{d}\right)={\prod}_{\boldsymbol{i}\in {\mathbf{\mathcal{M}}}_{\boldsymbol{d}}^{\boldsymbol{tr}}}{\prod}_{\boldsymbol{j}\in {\mathbf{\mathcal{M}}}^{\boldsymbol{tr}}\backslash {\mathbf{\mathcal{M}}}_{\boldsymbol{d}}^{\boldsymbol{tr}}}\mathbf{\Pr}\left({\hat{\boldsymbol{r}}}_{\boldsymbol{d}\boldsymbol{i}}>{\hat{\boldsymbol{r}}}_{\boldsymbol{d}\boldsymbol{j}}\right)\left[1-\Pr \left({\hat{\boldsymbol{r}}}_{\boldsymbol{d}\boldsymbol{i}}>{\hat{\boldsymbol{r}}}_{\boldsymbol{d}\boldsymbol{j}}\right)\right]$$

here $\boldsymbol{i}\boldsymbol{\in }{\mathbf{\mathcal{M}}}_{\boldsymbol{d}}^{\boldsymbol{tr}}$ indicates the known microbe-disease association pair (*i*, *d*) in training data and $\boldsymbol{j}\boldsymbol{\in }{\mathbf{\mathcal{M}}}^{\boldsymbol{tr}}\backslash {\mathbf{\mathcal{M}}}_{\boldsymbol{d}}^{\boldsymbol{tr}}$ represents the microbe-disease association pair (*j*, *d*) is unknown. We assume that the group preference is an overall preference score of a microbe group on a disease. If microbe-disease pair (*i*, *d*) is a known association but (*i*, *b*) is not, the group preference can be represented as:

11 $$\left(\mathbf{\mathcal{G}},\boldsymbol{d}\right)\succ \left(\mathbf{\mathcal{G}},\boldsymbol{b}\right),\mathbf{where}\ \boldsymbol{i}\in \mathbf{\mathcal{G}}\ \boldsymbol{and}\ \mathbf{\mathcal{G}}\subseteq {\mathbf{\mathcal{M}}}_{\boldsymbol{d}}^{\boldsymbol{tr}}$$

It can assume that the group preference of $\mathbf{\mathcal{G}}\subseteq {\mathbf{\mathcal{M}}}_{\boldsymbol{d}}^{\boldsymbol{tr}}$ on a disease *d* is probably stronger than the individual preference of microbe *i* on disease *b*. To learn the unified effect of both individual preference and group preference, we linearly combine them as follows:

12 $$\left(\mathbf{\mathcal{G}},\boldsymbol{d}\right)+\left(\boldsymbol{i},\boldsymbol{d}\right)\succ \left(\boldsymbol{i},\boldsymbol{b}\right)\;\mathrm{or}\;{\hat{\boldsymbol{r}}}_{\mathbf{\mathcal{G}}\boldsymbol{id}}>{\hat{\boldsymbol{r}}}_{\boldsymbol{ib}}$$

where ${\hat{\boldsymbol{r}}}_{\mathbf{\mathcal{G}}\boldsymbol{id}}=\boldsymbol{\rho} {\hat{\boldsymbol{r}}}_{\mathbf{\mathcal{G}}\boldsymbol{d}}+\left(\mathbf{1}-\boldsymbol{\rho} \right){\hat{\boldsymbol{r}}}_{\boldsymbol{id}}$ is the combined preference of individual preference ${\hat{\boldsymbol{r}}}_{\boldsymbol{id}}$ and group preference ${\hat{\boldsymbol{r}}}_{\mathbf{\mathcal{G}}\boldsymbol{d}}$ and parameter ***ρ*** controls the weight of two different preferences from 0 to 1. We equally set ***ρ*** to 0.5 in this study. In this way, we can define group Bayesian disease-oriented ranking (GBDR) analogously to how we define in Eq. (10) as follows:

13 $$\mathbf{GBDR}\left(\boldsymbol{i}\right)={\prod}_{\boldsymbol{d}\in {\mathbf{\mathcal{D}}}_{\boldsymbol{i}}^{\boldsymbol{tr}}}{\prod}_{\boldsymbol{b}\in {\mathbf{\mathcal{D}}}^{\boldsymbol{tr}}\backslash {\mathbf{\mathcal{D}}}_{\boldsymbol{i}}^{\boldsymbol{tr}}}\mathbf{\Pr}\left({\hat{\boldsymbol{r}}}_{\mathbf{\mathcal{G}}\boldsymbol{id}}>{\hat{\boldsymbol{r}}}_{\boldsymbol{i}\boldsymbol{b}}\right)\left[1-\Pr \left({\hat{\boldsymbol{r}}}_{\boldsymbol{i}\boldsymbol{b}}>{\hat{\boldsymbol{r}}}_{\mathbf{\mathcal{G}}\boldsymbol{id}}\right)\right]$$

where $\mathbf{\mathcal{G}}\subseteq {\mathbf{\mathcal{M}}}_{\boldsymbol{d}}^{\boldsymbol{tr}}$. $\boldsymbol{d}\boldsymbol{\in }{\mathbf{\mathcal{D}}}_{\boldsymbol{i}}^{\boldsymbol{tr}}$ means disease *d* has an interaction with microbe *i* in training data. Likewise, $\boldsymbol{b}\boldsymbol{\in }{\mathbf{\mathcal{D}}}^{\boldsymbol{tr}}\backslash {\mathbf{\mathcal{D}}}_{\boldsymbol{i}}^{\boldsymbol{tr}}$ means disease *b* has an unknown interaction with microbe *i*. For given two microbes *i* and *j*, the joint likelihood is simply approximated via multiplication like **GBDR**(**i**, **j**) **≈ GBDR**(**i**) **× GBDR**(**j**). Therefore, the overall likelihood for all microbes and all diseases can be formulated as:

14 $$\mathbf{GBDR}={\prod}_{\boldsymbol{i}\in {\mathbf{\mathcal{M}}}^{\boldsymbol{tr}}}{\prod}_{\boldsymbol{d}\in {\mathbf{\mathcal{D}}}_{\boldsymbol{i}}^{\boldsymbol{tr}}}{\prod}_{\boldsymbol{b}\in {\mathbf{\mathcal{D}}}^{\boldsymbol{tr}}\backslash {\mathbf{\mathcal{D}}}_{\boldsymbol{i}}^{\boldsymbol{tr}}}\Pr \left({\hat{\boldsymbol{r}}}_{\mathbf{\mathcal{G}}\boldsymbol{id}}>{\hat{\boldsymbol{r}}}_{\boldsymbol{i}\boldsymbol{b}}\right)\left[1-\Pr \left({\hat{\boldsymbol{r}}}_{\boldsymbol{i}\boldsymbol{b}}>{\hat{\boldsymbol{r}}}_{\mathbf{\mathcal{G}}\boldsymbol{id}}\right)\right]\kern1.25em$$

where $\mathbf{\mathcal{G}}\subseteq {\mathbf{\mathcal{M}}}_{\boldsymbol{d}}^{\boldsymbol{tr}}$. Given $\boldsymbol{\Theta} =\left\{{\boldsymbol{U}}_{\boldsymbol{i}}\boldsymbol{\in}{\mathbb{R}}^{\mathbf{1}\times \boldsymbol{nd}},{\boldsymbol{V}}_{\boldsymbol{d}}\boldsymbol{\in}{\mathbb{R}}^{\mathbf{1}\times \boldsymbol{nd}},{\boldsymbol{b}}_{\boldsymbol{d}}\in \mathbb{R},\boldsymbol{i}\boldsymbol{\in }{\mathbf{\mathcal{M}}}^{\boldsymbol{tr}},\boldsymbol{d}\boldsymbol{\in }{\mathbf{\mathcal{D}}}^{\boldsymbol{tr}}\ \right\}$ is a set of model parameters to be learned, one common way to estimate the model parameters is to minimize the log-likelihood function of GBDR as follows,

15 $$\underset{\boldsymbol{\Theta}}{\mathbf{\min}}-\frac{\mathbf{1}}{\mathbf{2}}\mathbf{\ln}\boldsymbol{GBDR}+\frac{\mathbf{1}}{\mathbf{2}}\mathbf{\mathcal{R}}\left(\boldsymbol{\Theta} \right).$$

We use stochastic gradient descent (SGD) algorithm to optimize the object function in Eq.(15). Before using the algorithm of SGD, a subset of microbes is randomly sampled to form a microbe group $\mathbf{\mathcal{G}}$. In this way, for each random sampling, it includes a microbe *i*, a disease *d*, a disease *b* and a microbe group $\mathbf{\mathcal{G}}$ where $\mathbf{i}\mathbf{\in}\mathbf{\mathcal{G}}$. The objective function in Eq. (15) can be written as:

16 $${\displaystyle \begin{array}{c}\mathbf{\mathcal{F}}\left(\mathbf{\mathcal{G}},\boldsymbol{i},\boldsymbol{d},\boldsymbol{b}\right)=-\mathbf{\ln}\left({\hat{\boldsymbol{r}}}_{\mathbf{\mathcal{G}}\boldsymbol{id}}-{\hat{\boldsymbol{r}}}_{\boldsymbol{ib}}\right)+\frac{{\boldsymbol{\alpha}}_{\boldsymbol{u}}}{\mathbf{2}}\sum_{\boldsymbol{j}\in \mathbf{\mathcal{G}}}{\left\Vert {\boldsymbol{U}}_{\boldsymbol{j}}\right\Vert}^{\mathbf{2}}+\frac{{\boldsymbol{\alpha}}_{\boldsymbol{v}}}{\mathbf{2}}{\left\Vert {\boldsymbol{V}}_{\boldsymbol{d}}\right\Vert}^{\mathbf{2}}\\ {}+\frac{{\boldsymbol{\alpha}}_{\boldsymbol{v}}}{\mathbf{2}}{\left\Vert {\boldsymbol{V}}_{\boldsymbol{b}}\right\Vert}^{\mathbf{2}}+\frac{{\boldsymbol{\beta}}_{\boldsymbol{v}}}{\mathbf{2}}{\left\Vert {\boldsymbol{b}}_{\boldsymbol{d}}\right\Vert}^{\mathbf{2}}+\frac{{\boldsymbol{\beta}}_{\boldsymbol{v}}}{\mathbf{2}}{\left\Vert {\boldsymbol{b}}_{\boldsymbol{b}}\right\Vert}^{\mathbf{2}}\\ {}\begin{array}{c}=\mathbf{\ln}\left[\mathbf{1}+\mathbf{\exp}\left(-{\hat{\boldsymbol{r}}}_{\mathbf{\mathcal{G}}\boldsymbol{id};\boldsymbol{ib}}\right)\right]+\frac{{\boldsymbol{\alpha}}_{\boldsymbol{u}}}{\mathbf{2}}\sum_{\boldsymbol{j}\in \mathbf{\mathcal{G}}}{\left\Vert {\boldsymbol{U}}_{\boldsymbol{j}}\right\Vert}^{\mathbf{2}}+\frac{{\boldsymbol{\alpha}}_{\boldsymbol{v}}}{\mathbf{2}}{\left\Vert {\boldsymbol{V}}_{\boldsymbol{d}}\right\Vert}^{\mathbf{2}}\\ {}+\frac{{\boldsymbol{\alpha}}_{\boldsymbol{v}}}{\mathbf{2}}{\left\Vert {\boldsymbol{V}}_{\boldsymbol{b}}\right\Vert}^{\mathbf{2}}+\frac{{\boldsymbol{\beta}}_{\boldsymbol{v}}}{\mathbf{2}}{\left\Vert {\boldsymbol{b}}_{\boldsymbol{d}}\right\Vert}^{\mathbf{2}}+\frac{{\boldsymbol{\beta}}_{\boldsymbol{v}}}{\mathbf{2}}{\left\Vert {\boldsymbol{b}}_{\boldsymbol{b}}\right\Vert}^{\mathbf{2}}\end{array}\end{array}}$$

where ${\hat{\boldsymbol{r}}}_{\mathbf{\mathcal{G}}\boldsymbol{id};\boldsymbol{ib}}={\hat{\boldsymbol{r}}}_{\mathbf{\mathcal{G}}\boldsymbol{id}}-{\hat{\boldsymbol{r}}}_{\boldsymbol{ib}}$, and ***α***_***u***_, ***α***_***v***_ and ***β***_***v***_ are the regularization weights ranging from 0.0001 to 0.1. ***U***_***j***_ ∈ *ℝ*^1 × *z*^ is the latent feature vector for microbe *j*, where z is the number of latent features. ***V***_***d***_ ∈ *ℝ*^1 × *z*^ and ***b***_***d***_ are disease *d*’s latent feature vector and bias values, respectively. We can then update the model parameters **Θ** as:

17 $$\boldsymbol{\Theta} =\boldsymbol{\Theta} -\boldsymbol{\upgamma} \frac{\boldsymbol{\partial}\mathbf{\mathcal{F}}\left(\mathbf{\mathcal{G}},\boldsymbol{i},\boldsymbol{d},\boldsymbol{b}\right)}{\boldsymbol{\partial}\boldsymbol{\Theta }}$$

where the learning rate **γ** is set to 0.01 in this study via parameter tuning. The learning process is repeatedly trained until it reaches the maximum iterations (default: 100). The predicted score of microbe *i* on disease *d* is calculated via ${\hat{\boldsymbol{r}}}_{\boldsymbol{d}\boldsymbol{i}}={\boldsymbol{V}}_{\boldsymbol{d}}^{\boldsymbol{T}}{\boldsymbol{U}}_{\boldsymbol{i}}+{\boldsymbol{b}}_{\boldsymbol{d}}$. The calculation procedure of GBDR is described by the pseudo-code in Algorithm 1.

Then we calculate ${\hat{\boldsymbol{r}}}_{\boldsymbol{di}}$ with the integrated microbe similarity *S*_*m*_ and disease semantic similarity *S*_*d*_. For an unknown disease-microbe pair (*d*_*i*_, *m*_*j*_), $\boldsymbol{d}^{\prime}\mathbf{\in}{\mathbf{\mathcal{D}}}_{{\boldsymbol{m}}_{\boldsymbol{j}}}^{\boldsymbol{tr}}$ means a set of diseases having associations with microbe *m*_*j*_ in training data and $\boldsymbol{m}^{\prime}\mathbf{\in}{\mathbf{\mathcal{M}}}_{{\boldsymbol{d}}_{\boldsymbol{i}}}^{\boldsymbol{tr}}$ indicates a set of microbes having associations with disease *d*_*i*_. Finally, the final prediction score of *d*_*i*_ on *m*_*j*_ could be calculated by adding the mean values as follows:

18 $${\hat{\boldsymbol{r}}}_{{\boldsymbol{d}}_{\boldsymbol{i}}{\boldsymbol{m}}_{\boldsymbol{j}}}+=\frac{{\boldsymbol{\alpha}}_{\boldsymbol{d}}}{\left|\boldsymbol{d}\hbox{'}\right|}{\sum}_{\boldsymbol{d}\hbox{'}\in {\mathbf{\mathcal{D}}}_{{\boldsymbol{m}}_{\boldsymbol{j}}}^{\boldsymbol{tr}}}{\boldsymbol{S}}_{\boldsymbol{d}}\left({\boldsymbol{d}}_{\boldsymbol{i}},{\boldsymbol{d}}^{\prime}\right)+\frac{{\boldsymbol{\alpha}}_{\boldsymbol{m}}}{\left|\boldsymbol{m}\hbox{'}\right|}{\sum}_{\boldsymbol{m}\hbox{'}\in {\mathbf{\mathcal{M}}}_{{\boldsymbol{d}}_{\boldsymbol{i}}}^{\boldsymbol{tr}}}{\boldsymbol{S}}_{\boldsymbol{m}}\left({\boldsymbol{m}}_{\boldsymbol{j}},{\boldsymbol{m}}^{\prime}\right)$$

where parameters ***α***_***d***_ and ***α***_***m***_ control the weights of *S*_*m*_ and *S*_*d*_ respectively. In this way, ${\hat{\boldsymbol{r}}}_{{\boldsymbol{d}}_{\boldsymbol{i}}{\boldsymbol{m}}_{\boldsymbol{j}}}$ is the predicted probability score of the unknown disease-microbe pair (*d*_*i*_, *m*_*j*_) ranging from − 1 to 1. The higher value ${\hat{\boldsymbol{r}}}_{{\boldsymbol{d}}_{\boldsymbol{i}}{\boldsymbol{m}}_{\boldsymbol{j}}}$, the higher probability of the potential association between disease *d* and microbe *j*. Then the model calculate $\hat{\boldsymbol{r}}$ for each unknown microbe-disease association. Finally, the potential microbe-disease associations can be predicted by ranking the predicted probability scores. The total time complexity of the proposed model is $O\left( Tnm\left|\mathbf{\mathcal{G}}\right|z\right)$.

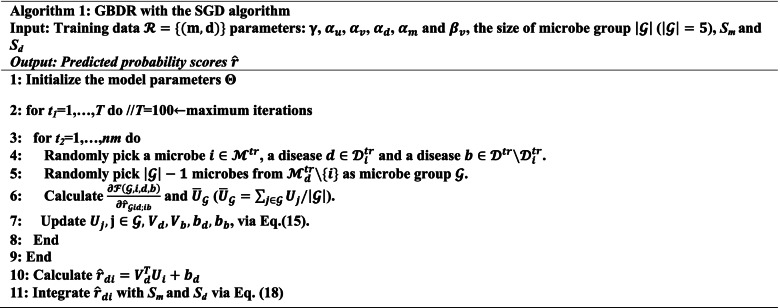



## Supplementary Information

**Additional file 1.** The case study of colorectal carcinoma.

**Additional file 2.** The prediction list of most likely pathogenic microbes is sorted by final prediction scores.

**Additional file 3.** Names of all investigated microbes and diseases, and known human microbe-disease associations obtained from HMDAD database.

## Data Availability

The datasets used and/or analysed in this study can be downloaded from the following public databases: the dataset of 16S rRNA partial or complete gene sequences from the National Center for Biotechnology Information (NCBI) repository (https://www.ncbi.nlm.nih.gov/); the dataset of MeSH descriptors from the Nation Library of Medicine (NLM) repository (https://www.nlm.nih.gov/mesh/meshhome.html); the dataset of known microbe-disease associations from the Human Microbe-Disease Association Database (HMDAD) repository (http://www.cuilab.cn/hmdad).

## Abbreviations

rRNA: ribosomal RNA
IBD: inflammatory bowel disease
OTUs: operational taxonomic units
LOOCV: leave-one-out cross validation
k-fold CV: k-fold cross validation
ROC: receiver operating characteristic
AUC: area under ROC curve
SVD: singular value decomposition
CF: collaborative filtering
CRC: colorectal carcinoma
NCBI: National Centre for Biotechnology Information
MeSH: Medical Subject Headings
NLM: Nation Library of Medicine
DAGs: Directed Acyclic Graphs
BLAST: Basic Local Alignment Search Tool
SGD: stochastic gradient descent
